# Genetic diversity and signatures of selection for heat tolerance and immune response in Iranian native chickens

**DOI:** 10.1186/s12864-022-08434-7

**Published:** 2022-03-22

**Authors:** Hojjat Asadollahpour Nanaei, Hamed Kharrati-Koopaee, Ali Esmailizadeh

**Affiliations:** Department of Animal Science, Faculty of Agriculture, Shahid Bahonar University of Kerman, 76169-133 Kerman, PB Iran

**Keywords:** Adaptation, Population genomics, Iranian indigenous chickens, Heat shock protein, Whole-genome sequence

## Abstract

**Background:**

Understanding how evolutionary forces relating to climate have shaped the patterns of genetic variation within and between species is a fundamental pursuit in biology. Iranian indigenous chickens have evolved genetic adaptations to their local environmental conditions, such as hot and arid regions. In the present study, we provide a population genome landscape of genetic variations in 72 chickens representing nine Iranian indigenous ecotypes (Creeper, Isfahan, Lari, Marand, Mashhad, Naked neck, Sari, Shiraz and Yazd) and two commercial lines (White Leghorn and Arian). We further performed comparative population genomics to evaluate the genetic basis underlying variation in the adaptation to hot climate and immune response in indigenous chicken ecotypes. To detect genomic signatures of adaptation, we applied nucleotide diversity (θπ) and *F*_ST_ statistical measurements, and further analyzed the results to find genomic regions under selection for hot adaptation and immune response-related traits.

**Results:**

By generating whole-genome data, we assessed the relationship between the genetic diversity of indigenous chicken ecotypes and their genetic distances to two different commercial lines. The results of genetic structure analysis revealed clustering of indigenous chickens in agreement with their geographic origin. Among all studied chicken groups, the highest level of linkage disequilibrium (LD) (~ 0.70) was observed in White Leghorn group at marker pairs distance of 1 Kb. The results from admixture analysis demonstrated evidence of shared ancestry between Arian individuals and indigenous chickens, especially those from the north of the country. Our search for potential genomic regions under selection in indigenous chicken ecotypes revealed several immune response and heat shock protein-related genes, such as *HSP70*, *HSPA9*, *HSPH1*, *HSP90AB1* and *PLCB4* that have been previously unknown to be involved in environmental-adaptive traits. In addition, we found some other candidate loci on different chromosomes probably related with hot adaptation and immune response-related traits.

**Conclusions:**

The work provides crucial insights into the structural variation in the genome of Iranian indigenous chicken ecotypes, which up to now has not been genetically investigated. Several genes were identified as candidates for drought, heat tolerance, immune response and other phenotypic traits. These candidate genes may be helpful targets for understanding of the molecular basis of adaptation to hot environmental climate and as such they should be used in chicken breeding programs to select more efficient breeds for desert climate.

**Supplementary Information:**

The online version contains supplementary material available at 10.1186/s12864-022-08434-7.

## Background

Domestic animals are excellent models for genetic studies of phenotypic evolution, due to strong phenotypic selection over many generations [1]. They have genetically evolved to adapt in diverse environmental conditions. Chickens (*Gallus gallus domesticus*), as the most extensively distributed of the poultries, have long been bred after its initial domestication in Southeast Asia, probably from the red junglefowl (*Gallus gallus*), around 9500 ± 3300 years ago [2]. Today, indigenous chickens are one of the most important sources of high-quality food in the livelihood of rural communities that well adapted to survive in their local conditions [3, 4]. Similarly, the native chicken populations of Iran are socially significant. They have been less artificially selected than commercial lines, which may harbour unique gene pools as a result of long-term adaptation to conditions prevailing in their ecosystem.

During recent decades, observed global climate data clearly display a warming trend in most part of the globe, resulting in a wide range of climatic impacts [5, 6]. Iran is located on Afro-Asian belt of deserts with 90% drylands extent, which climatically considered as a hot-arid region in the world [7, 8]. Indigenous chicken ecotypes of Iran are consequently raised from different parts of this land, which are well known for their tolerance to drought climate, low input regimes and also endemic diseases [9]. For example, the Creeper chicken is a native Iranian ecotype with small body mass (around 1 kg) that originated from Sistan and Baluchestan province, located in southeast of the country. The living environment of this ecotype belongs to the temperate desert climate zone with high solar radiation, high daytime temperatures, low rainfall (113 mm/year), frequent floating dust weather and high evaporation [10]. Since chickens with smaller body mass have a higher surface area to body weight ratio, the smaller body mass of Creeper chickens would help them to survive in hot and arid conditions. The Naked neck chicken is one another Iranian chicken ecotype that inhabit in northern parts of Iran. Previous studies have shown that the Naked neck chicken breeds have better growth performance in higher temperature than the normal breeds. Additionally, it has been suggested that the introduction of naked neck gene to reduce feather cover in the neck area of chickens that live in hot climates may be useful in breeding programs to improve the detrimental effects of heat tolerance [11].

In the last decade, some genomic studies in Iranian indigenous chickens based on limited microsatellite loci reported genetic variability between different ecotypes [12–14]. Only a few studies have been done to understand the genetic variation of different native ecotypes at the whole genome sequence level [15, 16]. Despite the fact that the Iranian indigenous chicken ecotypes, compared to commercial lines, may genetically have high thermo-tolerance capacity to survive in arid and semi-arid regions, there are lack of genomic studies related to heat tolerant traits in these birds [17]. Long-term natural selection in indigenous chickens leads to change in the allele frequency and thus favour adaptation as a product of evolution, however, commercial chickens have been bred through a series of massive artificial selection processes for meat production (broilers) or egg production (layers). The identification of loci under natural selection is of great interest in different research areas, which could increase understanding of the genetic mechanisms underpinning natural selection and adaptation to different local conditions [18].

In this study, for the first time, whole genome sequencing (WGS) data from most Iranian indigenous ecotypes (nine different populations) and two commercial lines (Arian and White Leghorn chickens, which are commonly used for meat and egg production, respectively) were used to characterize the genetic diversity, population structure and signatures of selection analysis. Results from comparative genomic analysis uncovered novel candidate genomic regions related to hot climate adaptation and immune response traits that could be under natural selection in indigenous chicken population. Our findings further provide new insights to better understanding the genetic relationship between indigenous chicken populations and commercial lines and will be helpful in the selection of superior native chickens.

## Methods

### Sampling and genomic DNA extraction

**Fig. 1:**
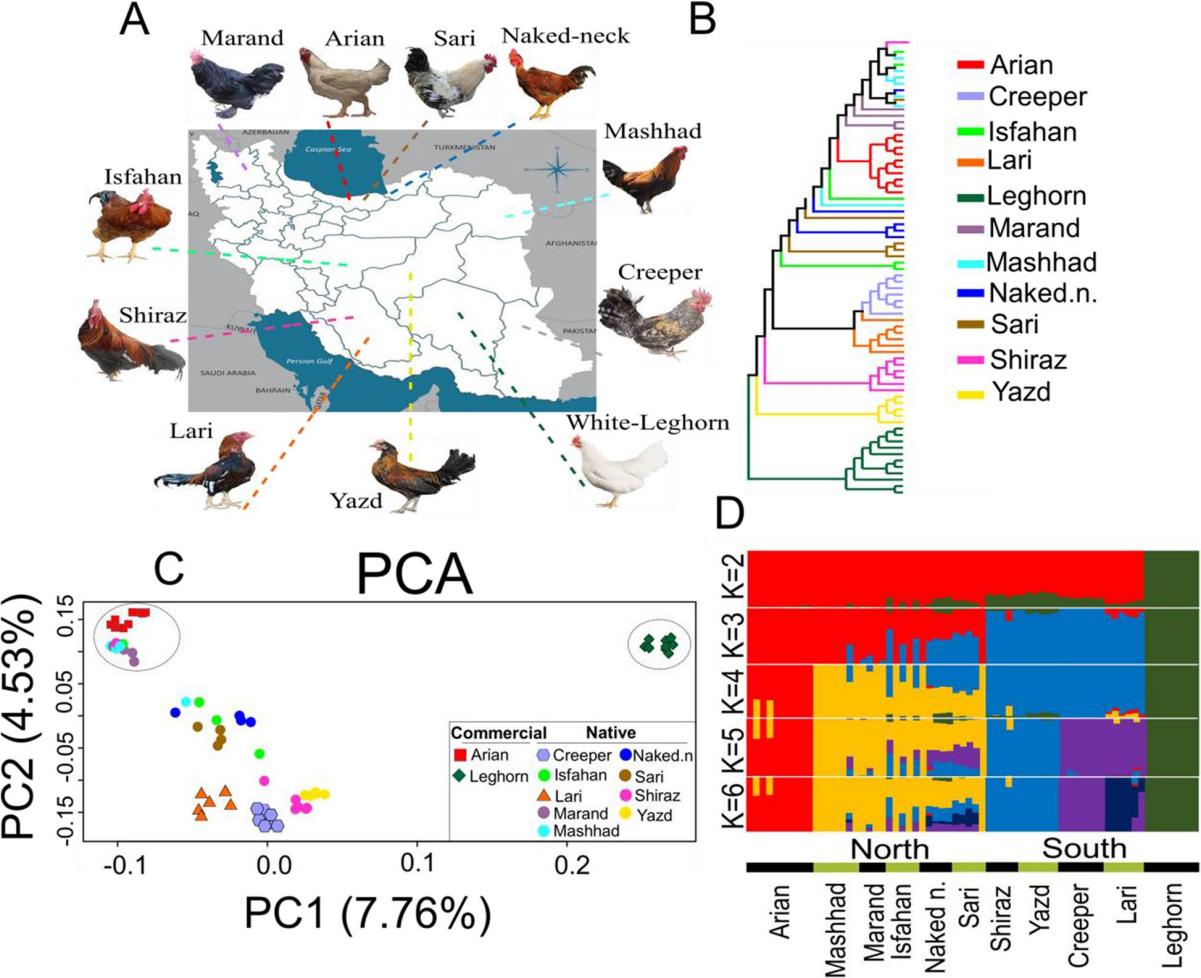
**A** Geographic locations of indigenous and commercial chicken populations. **B** Phylogenetic tree was built based on maximum-likelihood (ML) method. **C** Principal component (PC) analysis, PC 1 against PC 2. **D** ADMIXTURE model-based clustering analysis for each individual assuming different number of ancestral population (K = 2 to 6)

In this study, we sampled a total of 51 indigenous chickens from nine different ecotypes, including Creeper (*n* = 7), Isfahan (*n* = 5), Lari (*n* = 6), Marand (*n* = 4), Mashhad (*n* = 7), Naked neck (*n* = 5), Sari (*n* = 5), Shiraz (*n* = 7) and Yazd (*n* = 5). The samples were collected from different provinces as indicated in Fig. 1A (Additional file 1: Table S1). In general, the most dominant climate in Iran is arid which is characterized by long, dry and hot summer. For examples Yazd chicken ecotype lives in Yazd province, which has a hot desert climate region with a yearly precipitation of less than 65 mm [19]. Both Lari and Creeper ecotypes, that belong to the southern parts of Iran, are well adapted to the hot and dry climate conditions [20]. In addition, Naked neck chickens were collected from the north of Iran that genetically are more resistant to high temperatures [11]. Furthermore, 21 commercial chickens from two lines, White Leghorn (*n* = 11) and Arian (*n* = 10), were used in order to perform a comparison between different groups. For all studied individuals, venous blood samples (2 mL) were collected under the wing of the chickens for genomic DNA extraction method. No bird individuals died in this study, and all chickens stayed healthy after collecting blood samples. Total genomic DNA was isolated from whole blood samples using salting out protocol. The quality and quantity of the extracted DNA were tested by agarose gel (1%) electrophoresis and NanoDrop spectrophotometer analysis, and high-quality DNA samples were utilized for the subsequent whole genome resequencing.

### Re-sequencing of selected samples, quality checking and SNP calling

The individual genomes from nine indigenous chicken ecotypes (*n* = 51) and two commercial lines (*n* = 21) were sequenced on an Illumina Hiseq 2000 platform with a read length of 125 bp and ~ 10.2 × coverage (Additional file 1: Table S1). Read quality was evaluated using FastQC software (Version 0.4.2) (http://www.bioinformatics.babraham.ac.uk/projects/fastqc), and adapters and poor quality base pairs were removed by Trimmomatic software (version 0.36) [21]. Retained high-quality reads were then aligned to the reference chicken genome (GRCg6a: https://www.ncbi.nlm.nih.gov/assembly/GCF_000002315.5/) using Burrows Wheeler Aligner (BWA mem Version 0.7.10) [22]. The SAMtools software was used for converting SAM (.sam) and BAM (.bam) files, and for read sorting and indexing [23]. In order to reduce the risk of false positive variant calling, potential PCR duplicates were removed by Picard toolkit (http://broadinstitute.github.io/picard). To improve the alignment accuracy, base quality score recalibration (BQSR) and local realignment around indels were performed using Genome Analysis Toolkit (GATK) tools [24]. Final variants (single nucleotide polymorphism, SNPs) were called and filtered according to the GATK best practices recommendations. We further using Chicken dbSNP (Illumina chicken BeadChip and Affymetrix Axiom HD chicken genotyping array) preformed variant quality score recalibration (VQSR) by GATK package. By doing this step, we generated a recalibrated VCF file that includes the variation records with higher specificity. The haplotype phasing was performed using BEAGLE v.4.1 software [25]. All detected variants (~ 18.6 million SNPs) were then filtered to be supported by a minimum genotype quality of 40 and minimum mapping quality of 25. In order to avoid potential sequencing errors, all identified loci with more than two alleles and within clusters (> 3 SNP in a 10-bp window) were removed for the subsequent analyses [26]. After filtering out low-quality reads, around 14.56 million variants were retained for all individuals (Additional file 1: Table S2). A majority up to 11.45 million SNPs were observed in native group followed by 8.48 million in Arian group and 7.12 million in White Leghorn chickens. We observed 7.98 million SNPs shared between native and Arian group, which exceeded that shared between the native and White Leghorn group (5.9 million) (Additional file 2: Fig. S1).

### Population structure and admixture analyses

The maximum-likelihood (ML) method was applied to reconstruct phylogenetic tree. We first converted filtered VCF file into consensus FASTA files using vcf2fq in vcfutils.pl from Samtools [23], and then used FastTree 2 software [27] to generate tree. The online tool iTOL (https://itol.embl.de/) was used to visualize the topological structure.

Since linkage disequilibrium (LD) could modify the genetic structure analysis [28], the SNP dataset was first pruned for LD in PLINK (using PLINK option –indep-pairwise 50 10 0.1) [29]. The principal component analysis (PCA) and ADMIXTURE were carried out based on 6,562,417 SNPs after pruning for LD. We used genome-wide complex trait analysis (GCTA) based on SNP genotypes to assess genetic differences between all chicken groups [30]. To consider the possible genetic admixture between populations, we used the admixture model implemented in ADMIXTURE software, with an ancestor population (*K*) size ranging from 2 to 6 and 10,000 iterations for each run [31]. Additionally, haplotype sharing patterns were explored using the algorithm implemented in CHROMOPAINTER and fineSTRUCTURE softwares [32]. The decay of LD was calculated by Poplddecay software for different genetic distances (1, 3, 5, 15, 60 and 100 Kb) SNP pairs [33]. Runs of homozygosity (ROHs) were detected across all individual genomes via the “Runs of Homozygosity program” implemented in PLINK software with adjusted parameter (–homozyg-kb) [29]. For each chicken population, we identified all ROHs of length longer than 100 and 400 kb distances. We also calculated different genomic diversity parameters including observed heterozygosity (*H*o), expected heterozygosity (*H*e) and proportion of polymorphic SNPs (*P*n) using PLINK with the default settings. Additionally, we used the '–het' flag in PLINK to estimate the inbreeding coefficient (F) for each individual and each chicken group [29].

### Statistics to explore selective sweep regions

For the selective sweep analysis, two different methods were used to detect regions under selection. We calculated the genome-wide weighted *F*_ST_ [34], as it is a more precise measure of average genetic distance between groups with unequal samples [35]. The threshold of *F*_ST_ values were set to 0.210 and 0.195 (top 5% of empirical distribution for *F*_ST_ value), to determine outliers between indigenous chicken ecotypes and two commercial lines, White Leghorn and Arian groups, respectively. We then estimated nucleotide diversity θπ by using VCFtools (‐window‐pi 50,000 ‐‐window‐pi‐step 25,000) [36]. For the entire genome, sliding window analyses were performed with a window size of 50 kb and a step size of 25 kb. The average *F*_ST_ and log2 (θπ native/θπ commercial) values of SNPs in each window were calculated.

### Gene set enrichment and pathway analysis

To explore the possible pathways related to identified regions by the above-mentioned methods (*F*_ST_ and log2 θπ ratio), all candidate regions were annotated using the Variant Effect Predictor (VEP) available at (http://asia.ensembl.org/info/docs/tools/vep/index.html). Functional enrichment analysis, based on biological process Kyoto Encyclopedia of Genes and Genomes (KEGG) and Gene Ontology (GO) pathways, was then performed by using the ‘g:Profiler’ (https://biit.cs.ut.ee/gprofiler/) enrichment analysis tool, to uncover their biological functions. Finally, the *P*-value (*P* < 0.01) of the gene enrichment was corrected by Benjamini–Hochberg False Discovery Rate (FDR-BH).

## Results

### Population structure, runs of homozygosity and linkage disequilibrium decay

We conducted a series of classical analyses including phylogenetic assessments to identify population structure and the genetic relationship of different chicken groups. A phylogenetic tree for all individuals was constructed using ML method. Based on this tree (Fig. 1B), indigenous chicken individuals were separated far from White Leghorn samples but close to the Arian population. The PCA and ADMIXTURE results were also agreed with the phylogenetic tree (Figs. 1C and 1D). The early split between White Leghorn individuals and other chicken groups was indicated in the first component (PC1) that is consistent with phylogeny tree (Fig. 1C). The PC1 and PC2 accounted for 7.76% and 4.53% of the total genotypic variance, respectively. The Arian samples presented closer genetic distance to the indigenous individuals, thus nearly all samples from Mashhad and Marand ecotypes very tightly clustered close to this group, rather than other indigenous populations. Iranian chicken ecotypes could further be grouped into two groups, southern (Creeper, Yazd, Shiraz and Lari) and northern (Mashhad, Marand, Isfahan, Naked neck and Sari) populations. In order to better characterise the genetic relationship of indigenous chickens with other two commercial lines, the haplotype sharing patterns among individuals were estimated by ChromoPainter and fineSTRUCTURE software. FineSTRUCTURE is a model-based statistical algorithm that uses the coancestry matrix obtained from ChromoPainter to classify samples based on haplotype frequencies. The results are summarized into a “co-ancestry matrix”. Each row and each column indicates the result of expected coancestry between each chicken individual and other birds in the whole genome dataset. Dark colouring (blue) represents a high chunk count while the light colour (yellow) indicates a low chunk count. The colour bar on the right indicates the number of chunks (Additional file 2: Fig. S2). The results of painting algorithm provided substantial evidence of lower genetic similarity between indigenous chicken ecotypes and White Leghorn samples, rather than Arian group.

**Fig. 2:**
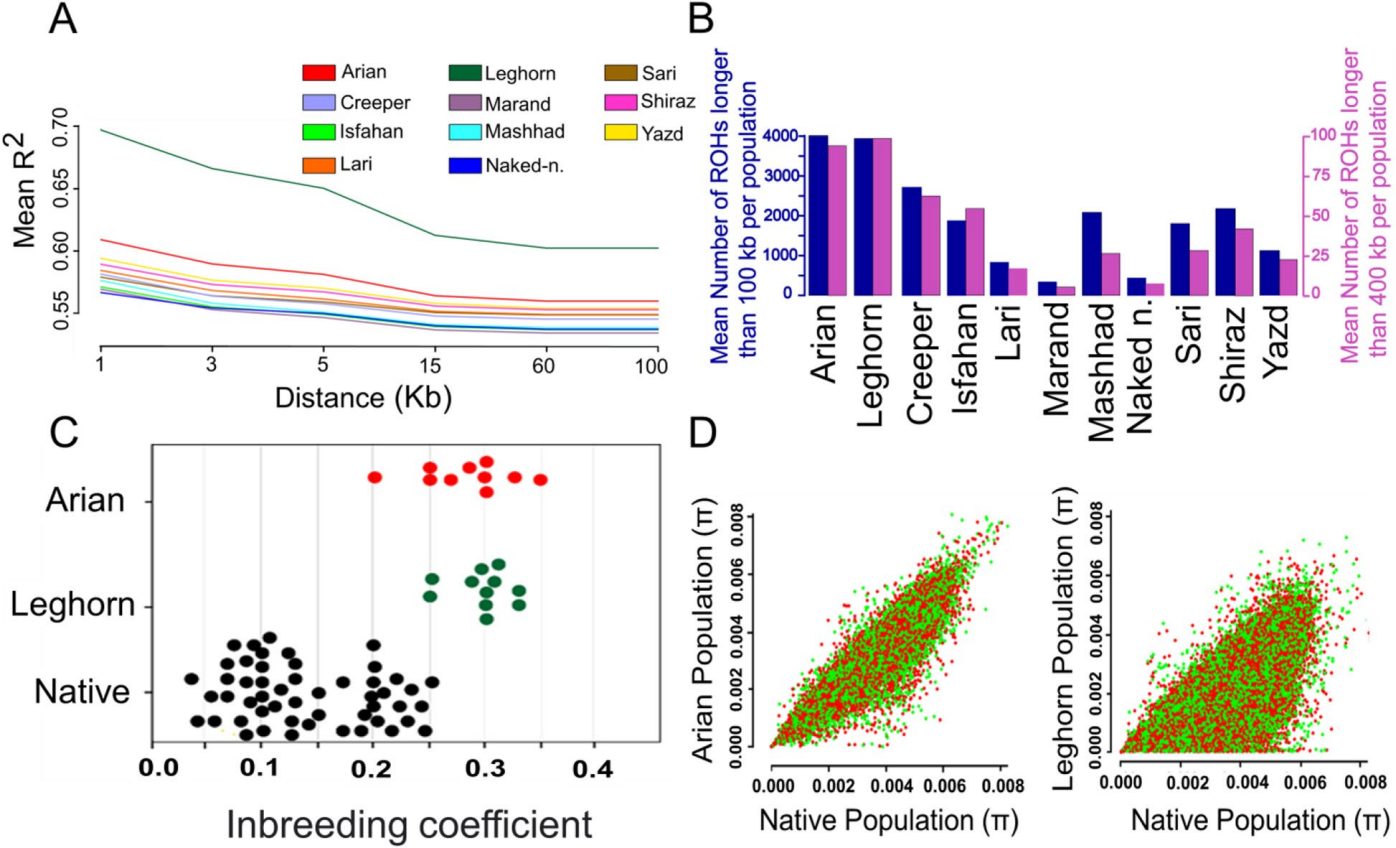
**A** Linkage disequilibrium (LD) decay. **B** Runs of homozygosity (ROH). **C** Inbreeding coefficient **F** for all chicken individuals. **D** Correlation of nucleotide diversity (θπ) (50-kb non-overlapping window) between indigenous chicken group (green) with two commercial lines (orange)

The clustering in ADMIXTURE (from K2 to K6) inferred the ancestry-component for all samples studied (Fig. 1D). The K = 2 splits the White Leghorn individuals from Arian and other Iranian chicken ecotypes. The K = 3 further distinguishes samples from north and south of the country, where the dominant component in the Arian population was detected in all ecotypes belonging to the north of Iran. When K = 4, with the lowest CV error (Additional file 2: Fig. S3), we found a separation between Arian population and chicken samples from the north of Iran (Fig. 1D). At K = 5, Lari and Creeper chickens divided from the remaining indigenous ecotypes in south of the country. In addition, plots with K = 5 and K = 6 clusters (Fig. 1D) suggest that some chicken ecotypes from north of Iran (mainly Sari and Naked neck) derive from genetic admixture between at least three populations. Ancestral proportions at K = 6 indicated that Yazd and Shiraz chickens were mainly assigned to the same cluster, while Lari and Creeper were separated from each other. Consequently, the results of admixture analysis demonstrated that Iranian ecotypes could be traced back to more than one single genetic source, suggesting multiple origins or origin events for native chickens. We then calculated the nucleotide diversity (θπ) within each chicken group and found that both two commercial groups have lower diversity than other indigenous ecotypes (Additional file 2: Fig. S4). The LD (R to the power of 2) decay rates between adjacent SNPs across the complete genome were determined to infer recent and historical effective population size (Ne). The results of LD decay analysis up to 100 kb are shown in Fig. 2A.

Our results showed a markedly higher level of LD across all genomic distances in both two commercial lines compare to all other indigenous ecotypes (Fig. 2A). As expected, the r^2^ values were the highest for all studied groups at marker pairs distance of 1 Kb (ranged from ~ 0.57 to 0.70, for Naked neck and White Leghorn, respectively) with a gradual decline with increasing physical distance between SNPs (up to 60 Kb) and then stable trend (> 60 Kb). The highest and lowest r^2^ values were observed in White Leghorn (0.61) and Marand (0.52) ecotypes at marker pairs distance of 100 Kb, respectively. We further found that from marker pairs distance of 1 Kb to ≤ 60 Kb, the decay of LD was more rapid in White Leghorn group than other birds, reaching an average r^2^ values from 0.70 to 0.61 (Fig. 2A). Focusing on the native ecotypes, higher r^2^ values across all genomic distances were found for Yazd ecotype that is genetically less mixed with other chicken groups (Fig. 1D), and dwelled in southern parts of the country. While the lower r^2^ values were found in Marand ecotype, that seems genetically admixed with at least one native chicken group (creeper ecotype) (Fig. 1D). We then calculated ROH at different cut-off lengths among studied groups. The majority of the detected ROHs were identified < 0.4 Mb for all chickens (Additional file 1: Table S3). Compared to the native ecotypes, commercial chickens showed a higher number of short to medium sized ROHs (0.1 to 0.4 Mb), with the highest number found in the White Leghorn (*n* = 5035) followed by Arian chickens (*n* = 4974) (Fig. 2B). The ROH segments varied in total numbers among native ecotypes, ranging from 425 to 3111 for Marand and Creeper, respectively. The mean number of detected ROHs longer than 100 Kb for all studied groups ranged from 362 to 4012 for Marand and White Leghorn, respectively. We also checked the distribution of long ROH (ROH size > 1 Mb) among groups and birds. As the results showed, except for three native ecotypes (Creeper, Naked neck and Yazd), no other chicken groups had ROH longer than 1 Mb in length (Additional file 1: Table S3). Long ROHs are likely to be due to recent inbreeding, because they have not yet been broken up by recombination, while short and medium sized ROHs are more likely the results of selection for a long period of time and more ancient inbreeding. We further estimated inbreeding coefficients (F) per each individual (ranged from ~ 0.04 to 0.37 for Naked neck and Arian, respectively) and chicken group (ranged from 0.07 to 0.36 for Naked neck and White Leghorn, respectively) (Fig. 2C and Additional file 1: Table S4). The maximum and minimum levels of average inbreeding coefficient for native ecotypes were 0.21 and 0.07, for Shiraz and Naked neck ecotypes, respectively. The results showed that all native ecotypes had lower inbreeding coefficient than commercial chicken populations. We then calculated the genetic diversity parameters in all chicken populations using whole-genome SNP data.

Our findings showed that indigenous chicken ecotypes have higher *Ho* values (average ~ 0.194) than both commercial groups (0.124 and 0.145, for White Leghorn and Arian, respectively). In all studied chicken groups, *P*n ranged from 0.634 (White Leghorn) to 0.912 (Marand), with a mean of 0.768 (Additional file 1: Table S4). Our finding from estimating the patterns of genetic divergence, as a function of θπ, between indigenous chickens and other two commercial populations revealed a strong genetic correlation between Iranian indigenous chicken group and Arian samples, which also could be due to their shared ancestry (Fig. 2D).

### Scanning of highly differentiated genomic regions

**Fig. 3:**
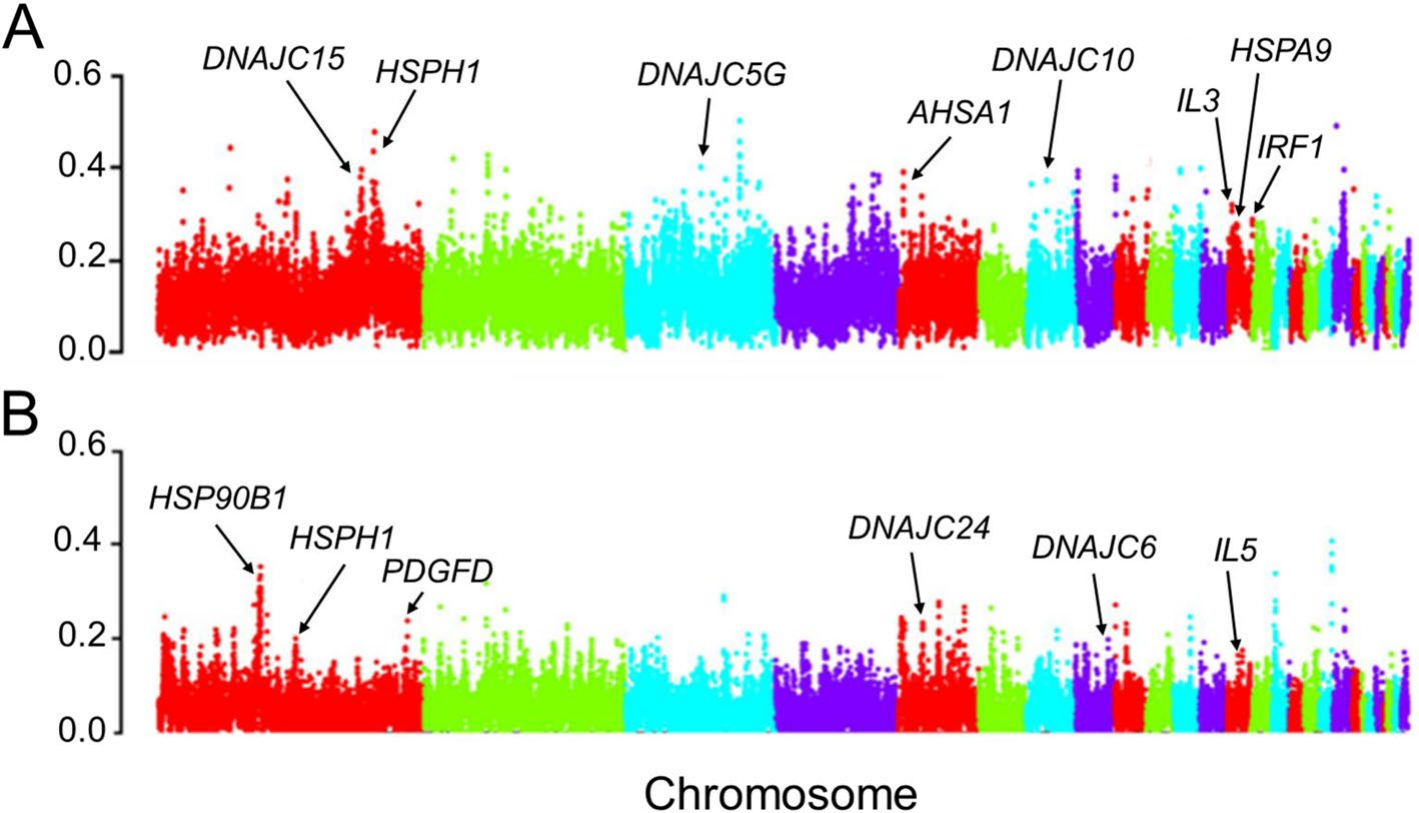
Genomic landscape of population differentiation by *F*_ST._ **A** Indigenous group versus White-Leghorn chickens. **B** Indigenous group versus Arian chickens**.** Non-coding DNA regions were not labeled

Our results revealed high levels (mean weighted *F*_ST_ > 0.6) of genetic differentiation between both commercial lines and indigenous chickens (Fig. 3).

The comparative genomics results between indigenous chicken ecotypes and White Leghorn group revealed a total of 853 and 309 protein-coding genes in windows with high *F*_ST_ (5% cutoff) and log2 θπ ratio values (1% cutoff), respectively (Additional file 1: Table S5 and Table S6). In addition, our results from selection signature statistics between indigenous chicken group and Arian samples revealed a total of 763 and 205 candidate genes in the top windows for *F*_ST_ (5% cutoff) and log2 ratio of θπ tests (1% cutoff), respectively (Additional file 1: Table S7 and Table S8; Additional file 2: Fig. S5). For further functional analysis, those genomic regions identified above were annotated to the corresponding genes and biological pathways to investigate the potential genetic mechanisms associated with different traits in chicken (Additional file 1: Tables S9-S12). The results showed several candidate genes that have previously been reported to associate with heat tolerance and immune-related traits (such as; *HSPA9*, *HSPH1*, *HSP70*, *HSP90AB1*, *NBEA*, *IL5*, *IL3* and *IRF1*) between native ecotypes and White Leghorn genomes (Table 1). KEGG mapping and gene set enrichment analysis (GSEA) identified some significantly enriched categories related with heat tolerant and immune response traits such as “abnormality of the immune system” (HP:0,002,715), “activation of MAPKK activity” (GO:0,000,186) and “phosphatidylinositol phosphate binding” (GO:1,901,981) (Additional file 1: Table S9 and Table S10). Moreover, comparative genomic results between native ecotypes and Arian genomes, showed some enriched categories, “MAPK signaling pathway” (KEGG:04,010) and “Deficiency of N-acetylglucosamine-1-phosphotransferase” (HP:0,003,264) (Additional file 1: Table S11 and Table S12), and genes (such as *HSP90B1*, *HSPH1*, *PLCB4* and *IL5*) that are involved in heat shock tolerance and/or immune activity related traits (Table 1).

**Table 1:** Candidate genes putatively selected by two statistical methods (*F*_ST_ and log_2_ θπ ratio and) affecting heat stress and immune responses traits in indigenous chicken ecotypes

Selected comparative model	Statistical-Method	Gene	Chr.*	Summary of gene function
Native ecotypes – White-Leghorn line	*F*_ST_ (top 5%)	*HSPA9*	13	Heat stress [37]
		*AHSA1*	5	Heat stress [38]
		*HSPH1*	1	Heat stress [39, 40]
		*DNAJC10*	7	Heat stress [41]
		*DNAJC6*	8	Heat stress [42]
		*DNAJC15*	1	Heat stress [41]
		*DNAJC5G*	3	Heat stress [41]
		*DNAJA4*	10	Heat stress [41]
		*IL3*	13	Immune response [43, 44]
		*IL13*	13	Immune response [43, 44]
		*IRF1*	13	Immune response [45]
	log2(θπ·Native/θπ·Commercial) (top 1%)	*HSP70*	5	Heat stress [43, 46–48]
		*HSPD1*	7	Heat stress [37, 49]
		*HSPH1*	1	Heat stress [39, 40]
		*NBEA*	1	Heat stress [50]
		*HSP90B1*	1	Heat stress [37, 51]
		*HSP90AB1*	3	Heat stress [52]
		*HSPA5*	17	Heat stress [53]
		*HSPB1*	19	Heat stress [53]
		*HSPA8*	24	Heat stress [53]
		*IRF1*	13	Immune response [45]
		*IL5*	13	Immune response [43, 44]
		*IL3*	13	Immune response [43, 44]
		*DNAJC15*	1	Heat stress [41]
Native ecotypes- Arian line	*F*_ST_ (top 5%)	*HSP90B1*	1	Heat stress [37, 51]
		*HSPH1*	1	Heat stress [39, 40]
		*DNAJC24*	5	Heat stress [41]
		*DNAJC6*	8	Heat stress [42]
		*IL5*	13	Immune response [43, 44]
	log2(θπ·Native/θπ·Commercial) (top 1%)	*HSPH1*	1	Heat stress traits [39, 40]
		*PLCB4*	3	Immune response and heat stress [49, 54, 55]
		*IRF1*	13	Immune response [45]

^*^Chromosome

## Discussion

### Genetic diversity and population structure

Information about population structure and genetic diversity among native chicken ecotypes is of fundamental importance for genetic improvement, understanding of environmental adaptation as well as for conservation and sustainable management programs [56]. Our study set out to analyze patterns of Iranian indigenous chicken genetic variation by sequencing 51 complete genomes from nine indigenous populations and comparing them to the genomes of two different (*n* = 21) commercial lines.

Iranian indigenous chickens are typically kept by smallholder farmers under extensive management systems and without controlled breeding programs [9]. In line with most previous studies, we found that the indigenous chicken ecotypes harbor higher levels of genetic diversity, as compared with the commercial lines, which could be due to inherent traditional breeding practices of natural and random mating of indigenous chickens [57, 58]. The estimated *Ho* values (ranged from 0.181 to 0.211) for native chicken ecotypes in the present study were relatively similar to those reported in native chicken ecotypes in Thailand and Jordan [59–61], but lower than Indian and African native ecotypes [62–64]. We observed no significant differences between *H*o and *H*e values in all chicken ecotypes. However, we found lower *H*o (ranged from 0.124 to 0.145) than *H*e values (ranged from 0.168 to 0.192) in both two commercial lines, showing a departure from Hardy–Weinberg equilibrium and therefore possibility of selection, non-random mating and/or inbreeding pressure within populations [65]. We further observed that among two commercial groups, White Leghorn chickens had the lowest genetic diversity (*H*o = 0.124), which is in agreement with previous studies [66] and could be attributed to the intense artificial selection over a short period of time and breed-formation practices [67].

Knowledge about the patterns of LD decay can provide important insights into population history, demographic processes and breeding systems [68, 69]. The LD estimates decreased with increasing genomic distance of SNP pairs, which was consistent with previous studies in chicken and livestock animals [70, 71]. We found that all of the studied indigenous ecotypes followed the same pattern of decrease in LD as the genomic distance increased, however more rapid decrease in LD over increasing genomic distance was observed in White leghorn chickens (Fig. 2A).

The r^2^ values obtained in this study for native ecotypes were in the ranges of 0.52 to 0.57 at marker pairs distance of 100 Kb, which agreed with those reported for Chinese and Canadian native chicken populations [70, 72]. We further found a markedly higher level of LD across all genomic distances in White Leghorn chickens followed by Arian group (Fig. 2A). The high level of LD in commercial chicken lines could be a consequence of higher level of inbreeding and extensive artificial selection for desired traits in the breeding programs [73]. In addition, quantifying the ROH per each studied group revealed the lower level of ROH in all indigenous populations compared with both two commercial chicken lines (Fig. 2B), that is in line with previous reports using microarray [74] and whole genome sequencing data [75]. Our results revealed that both length and number of ROHs varied among chicken ecotypes (Fig. 2B, Additional file 1: Table S3). The results from population genetic analyses, including phylogeny based on haplotype patterns, admixture (K = 2 and K = 3), and PCA, have collectively demonstrated the possibility of shared genetic ancestry between Arian line and native ecotypes, especially those chicken ecotypes from the north of country (Figs. 1B, 1D and 2D). It seems likely that the local chicken breeders, especially in the north of the country, have been focused on growth rate and meat production traits through cross-breeding of native ecotypes with Arian broiler lines to improve the genetic pool of local stock birds. To support this hypothesis, our results from population clustering programs such as PCA and Admixture, as well as estimating the patterns of genetic divergence show those native chicken ecotypes belong to the north of the country, where the companies related to exotic commercial strains such as viz (a pure Arian broiler line, which has a 78% share of the day-old chick market) established in the early 1980s [76], display low genetic distances with Arian group.

### The adaptation of indigenous chickens to environmental stresses

The native ecotypes are more likely to be a good model for exploring the genetic mechanisms underlying adaptation to local cultural conditions and disease. To investigate this, all indigenous chickens were combined and compared to two different commercial lines to uncover loci subjected to natural selection over a long number of generations. Previous reports showed that the heat tolerance negatively influences the health and production of livestock species, and today the genetic selection for heat-tolerant is a challenge in poultry industry [77–79]. Majority of studies have shown that there is genetic variation in performance of chicken populations under heat tolerance conditions [80, 81]. Therefore, identification and selection genes for heat tolerance is likely to offer a promising and long-term solution to address issues relating to this problem. Results from the detection of selection signatures between White Leghorn and indigenous chicken group, using two different statistical methods, revealed several putative candidate genes in the high-confidence selection regions (highest 5% *F*_ST_ and 1% log2 θπ ratio values) that may be involved in heat tolerance and immune response traits (Table 1). In our broad spectrum analysis, we found different heat shock protein (HSP) genes such as; *HSP70, HSPH1*, *HSPD1*, *HSP90AB1*, *HSPB1* and *HSPA8* to be probably associated with heat tolerance related traits in Iranian native chicken (Table 1 and Fig. 3A). HSPs are a large family of highly homologous chaperone proteins that are induced when cells are exposed to temperatures above their optimum for growth [82–85]. These proteins protect essential cell components from various types of harmful damages and also facilitate cellular recovery. *HSP70*, as one of the most important members of the HSP family, is located on chicken chromosome (GGA) 5 (53.058–53.060 MB). The expression/activity of this gene in response to different thermal challenges has been extensively investigated in chicken and mammals [46, 86, 87]. Previous studies have found associations of the *HSP70* variants with the survival ability of cells affected by heat tolerance. It was reported a silent mutation in coding region of *HSP70* could possibly be used as a heat tolerance marker in indigenous and commercial chickens [47]. The gene expression analysis showed that the expression level of chicken *HSP70* mRNA in the heart and liver of young White Leghorn was positively correlated with body temperature [88]. Most recently, the substitution of T with C in exon 1 (52.784621- 52.784671 Mb) of this gene was also suggested as a candidate marker for heat stress tolerance in breeding program of Dandarawi line chickens [89].

We further found some candidate genes on different chromosomes to be potentially involved in chicken immune system. For example, we identified a group of interleukin (*IL3*, *IL13*, *IL5*) and interferon (*IRF1*) genes that are responsible in immune response. Interleukins are a group of cytokines that expressed by leukocytes [90]. Several investigations attempt to explain the role of interleukins in immune system of chickens [91–93]. This finding is in agreement with previous studies that claimed genomic regions under selection in native chickens display significant enrichment for immune function genes [94, 95].

We then analyzed signatures of selection between Arian group and indigenous chicken ecotypes. Within the regions with higher values of *F*_ST_ (top 0.05) and reduced nucleotide diversity (top 0.01 of log2 θπ ratio values), we found some candidate genes associated with heat tolerance (such as; *HSPH1*, *HSP90B1*) and oxidative stress response (eg *PLCB4*) (Table 1 and Fig. 3B). HSPH1 (also called *Hsp105* or *Hsp110*) is a high molecular weight protein belonging to the HSP family that functions in cellular stress response and apoptosis pathways [96]. This protein has previously been shown to cooperate with HSP70 and HSP40 to prevent aggregation of misfolded proteins [97]. High level expression of *HSPH1* gene was also observed in chicken macrophage-like cell line under acute heat tolerance [39]. Previous global gene expression study has also showed that the *HSPH1* gene is differentially expressed in liver, brain and leg muscle of broiler chickens that exposed to heat tolerance [40]. Furthermore, the haplotype network of the *HSPH1* selected region (Chr1: 176.34–176.36 Mb) showed that the indigenous Iranian chicken haplotypes are mostly identical at all of the SNPs across this gene (Additional file 2: Fig. S6). PLCB4 is another protein coding gene that has important roles in regulating immune defense, energy metabolism and oxidative stress response in different animal species [54, 55]. Heat tolerance causes excessive oxygen consumption in organisms, which lead to optimized oxygen delivery by increasing blood flow in circulation [48]. Most recently, the potential roles of this gene in the MAPK signaling pathway, which is an upstream regulator of melanogenesis and melanoma angiogenesis, was described in detail in one experimental study on local chickens that live in tropical climates [98].

## Conclusions

This study represents the first attempt to investigate the genetic diversity and population structure of most Iranian indigenous chicken ecotypes by using whole-genome sequencing data. We estimated and compared several genetic diversity parameters such as LD, ROH, fixation index (*F*ST) and gene diversity (*H*e, *H*o and *P*n) between nine Iranian indigenous chicken ecotypes and two commercial lines. Multiple lines of evidence suggest that there is a high level of genetic distance between native chicken ecotypes and White Leghorn group. However, we observed a relatively low genetic variability between Arian group and native chicken ecotypes, especially those from northern parts of the country. We further found relatively high levels of LD decay and ROH in both two commercial lines, which could be a consequence of artificial selection for desired traits such as egg or meat production performances. In addition, we detected several candidate genes associated with adaptation to arid environment, immune response activation and parasite resistance in Iranian indigenous chicken ecotypes. Our finding will help improve our understanding of the mechanisms and identify the targets of selection in indigenous chickens that live in desert regions and also facilitate future genome wide association studies and investigations into genomic targets of selection [99].

## Supplementary Information

**Additional file 1:** **Table S1:** Sample information for each chicken (72 individuals) used in this study. **Table S2.** Chromosome-length and number of detected variants before and after filtering. **Table S3.** Mean number of ROHs longer than different classes (Kb).**Table S4.** Summary  diversity results. Proportion of polymorphic SNPs (PN ), Observed heterozygosity (Ho), Expected heterozygosity (He), and average inbreeding coefficient (F) for each studied group. Table S5: Positively selected genes (top %5) identified between indigenous group and White-Leghorn identified by FST method. **Table S6.** Positively selected genes identified by top 1% highest log2 (θπ·Native-ecotypes/θπ·White-Leghorn). **Table S7.** Positively selected genes (top %5) identified by FST method between indigenous group and Arian. Table S8: Positively selected genes identified by top 1% highest log2 (θπ·Native-ecotypes/θπ·Arian). **Table S9. **Overrepresented GO categories among genes showing high Fst values between indigenous group and  White-Leghorn. **Table S10. **Overrepresented GO categories among genes showing high log2 (θπ·Native-ecotypes/θπ·White-Leghorn). **Table S11.** Overrepresented GO categories among genes showing high Fst values (indigenous group versus Arian). **Table S12.** Overrepresented GO categories among genes showing high log2 (θπ·Native-ecotypes/θπ·Arian).

**Additional file 2:** **Figure S1.**Venndiagrams summarize unique and common variants among groups. **Fig. S2.** Theheat map of ChromoPainter’s coancestry matrix. Each row corresponds to therecipient genomes and columns represent the donor individuals. **Fig. S3.** Cross validationerror (CV) plot from ADMIXTURE. **Fig. S4.** Box plots of nucleotide diversity, calculated in 50kb sliding window with 20 kb increments across the genome. **Fig. S5. **Genomic regions with selection signals in nativechickens by π method. (A) Indigenousgroup versus Arian chickens. (B) Indigenous group versus White Leghornchickens. **Fig. S6.** Haplotype network based on pairwise differenceswithin the selective sweep region (Chr1: 176.34-176.36) in*HSPH1*.

## Data Availability

The whole genome sequencing data for all chicken individuals generated in this study have been deposited at NCBI SRA Database with accession code: PRJNA807738 or accessible through https://www.ncbi.nlm.nih.gov/bioproject/ PRJNA807738. The NCBI accession numbers used in this study can be found in Additional file 1: Table S1. Furthermore, the whole genome sequencing data, BAM files, and genotypes (VCF) have been submitted in the ChickenSD database (http://bigd.big.ac.cn/chickensd/).

## Abbreviations

BAM: Binary Alignment MAP
BWA: Burrows-Wheeler Aligner
*F*_ST_: Fixation index
LD: Linkage disequilibrium
GATK: Genome Analysis Toolkit
GO: Gene ontology
Θπ: Nucleotide diversity
PCA: Principle component analysis
ROH: Runs of homozygosity
SNP: Single-nucleotide polymorphism
GCTA: Genome-wide complex trait analysis
