# Correction: Genetic diversity and signatures of selection for heat tolerance and immune response in Iranian native chickens

**DOI:** 10.1186/s12864-022-08579-5

**Published:** 2022-05-24

**Authors:** Hojjat Asadollahpour Nanaei, Hamed Kharrati-Koopaee, Ali Esmailizadeh

**Affiliations:** Department of Animal Science, Faculty of Agriculture, Shahid Bahonar University of Kerman, Kerman, PB 76169-133 Iran

**Correction: BMC Genomics 23, 224 (2022)**

**https://doi.org/10.1186/s12864-022-08434-7**

Following publication of the original article [1], it was reported that the authors had not cited Wang et al. [2]. Therefore, the ‘Methods’ section, ‘Availability of data and materials’ declaration and the ‘References’ are incomplete and should be revised as described below.

In the ‘Re-sequencing of selected samples, quality checking and SNP calling’ sub-section the sentence highlighted in bold has been added:

“The individual genomes from nine indigenous chicken ecotypes (n=51) and two commercial lines (n=21) were sequenced on an Illumina Hiseq 2000 platform with a read length of 125 bp and~10.2×coverage (Additional file 1: Table S1). **Whole-genome sequencing of the samples using the next-generation sequencing technique was conducted within the framework of the Global Chicken Genome Project (****http://chicken.ynau.edu.cn/index/about/index.html****) led by Kunming Institute of Zoology, Chinese Academy of Sciences (KIZ-CAS) and described by Wang et al.** [2, 3].”

The ‘Availability of data and materials’ declaration has been revised.

The original declaration stated: “The whole genome sequencing data for all chicken individuals generated in this study have been deposited at NCBI SRA Database with accession code: PRJNA807738 or accessible through https://www.ncbi.nlm.nih.gov/bioproject/ PRJNA807738. The NCBI accession numbers used in this study can be found in Additional file 1: Table S1. Furthermore, the whole genome sequencing data, BAM files, and genotypes (VCF) have been submitted in the ChickenSD database (http://bigd.big.ac.cn/chickensd/).”

The revised declaration should read: “The datasets used for the current study were from Wang et al [2]. The raw sequencing data, alignment BAM files, and genotypes (VCF) are available in the ChickenSD database (http://bigd.big.ac.cn/chickensd/)“.

Finally, Additional file 1: Table S1 has been updated and is provided in this Correction article.

The original article [1] has been updated.

## Supplementary Information

**Additional file 1. Table S1**: Sample information for each chicken (72 individuals) used in this study.

## References

[1] Asadollahpour Nanaei H, Kharrati-Koopaee H, Esmailizadeh A (2022). Genetic diversity and signatures of selection for heat tolerance and immune response in Iranian native chickens. BMC Genomics.

[2] Wang MS, Thakur M, Peng MS, Jiang Y, Frantz LAF, Li M (2020). 863 genomes reveal the origin and domestication of chicken. Cell Res..

[3] Wang MS, Zhang JJ, Guo X, Li M, Meyer R, Ashari H (2021). Large-scale genomic analysis reveals the genetic cost of chicken domestication. BMC Biol..

