# Supplementary material for: Genetic diversity and signatures of selection for heat tolerance and immune response in Iranian native chickens
Source: BMC Genomics. 2022 Mar 22;23:224. doi: 10.1186/s12864-022-08434-7 (PMC8939082; doi:10.1186/s12864-022-08434-7)

Fig. S1.Venn diagrams summarize unique and common variants among groups.


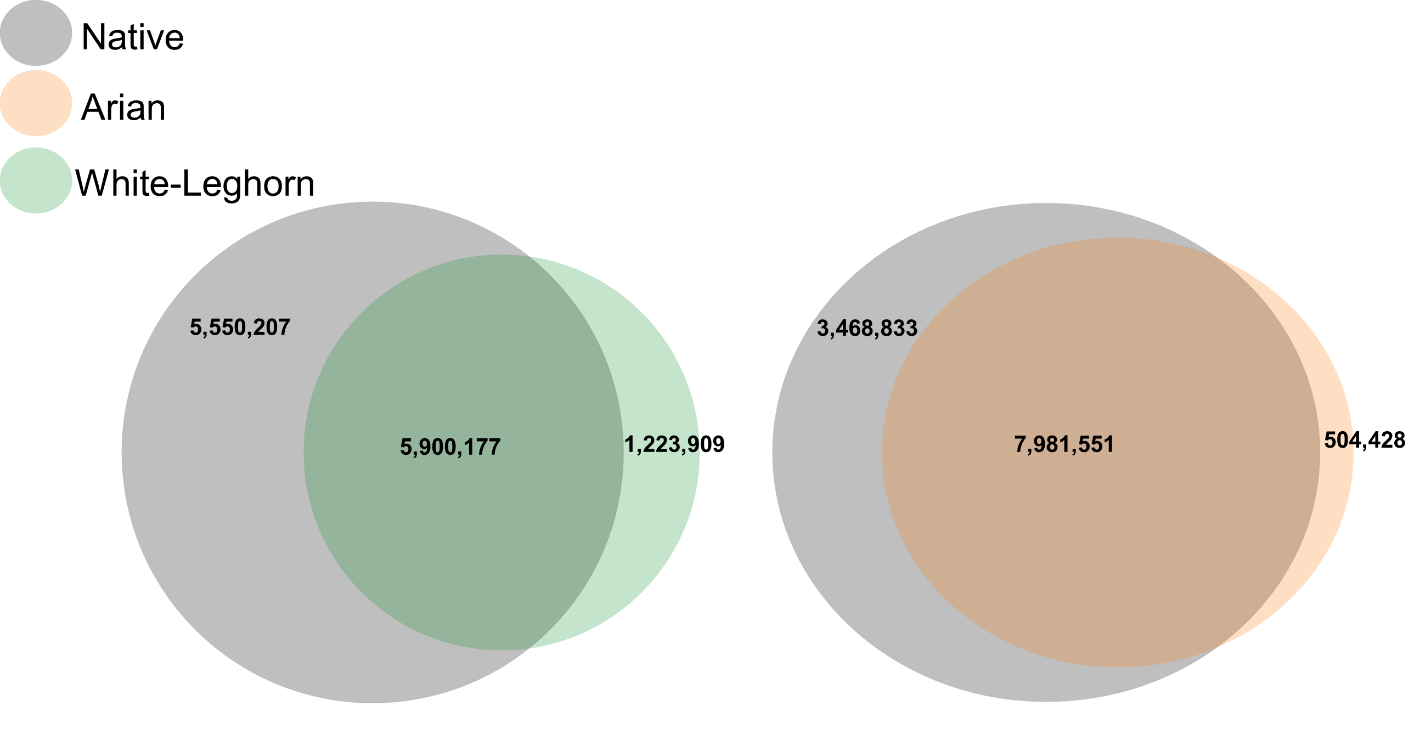


Fig. S2: The heat map of ChromoPainter’s coancestry matrix. Each row corresponds to the recipient genomes and columns represent the donor individuals.


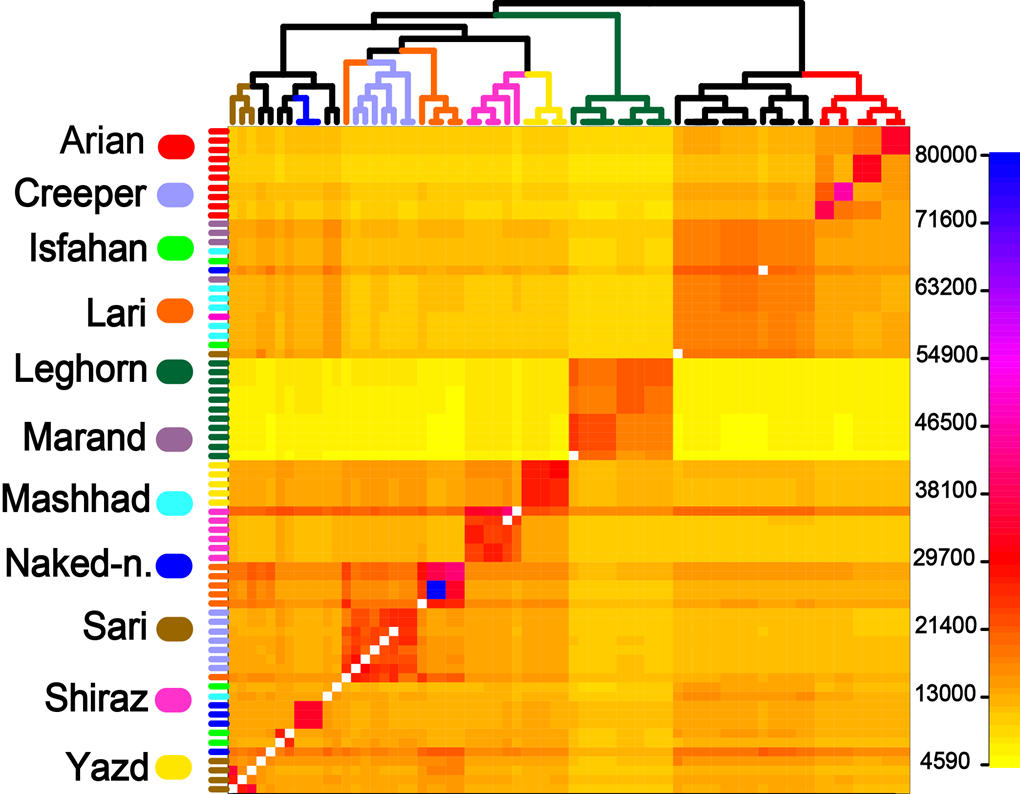


Fig. S3: Cross validation error (CV) plot from ADMIXTURE.


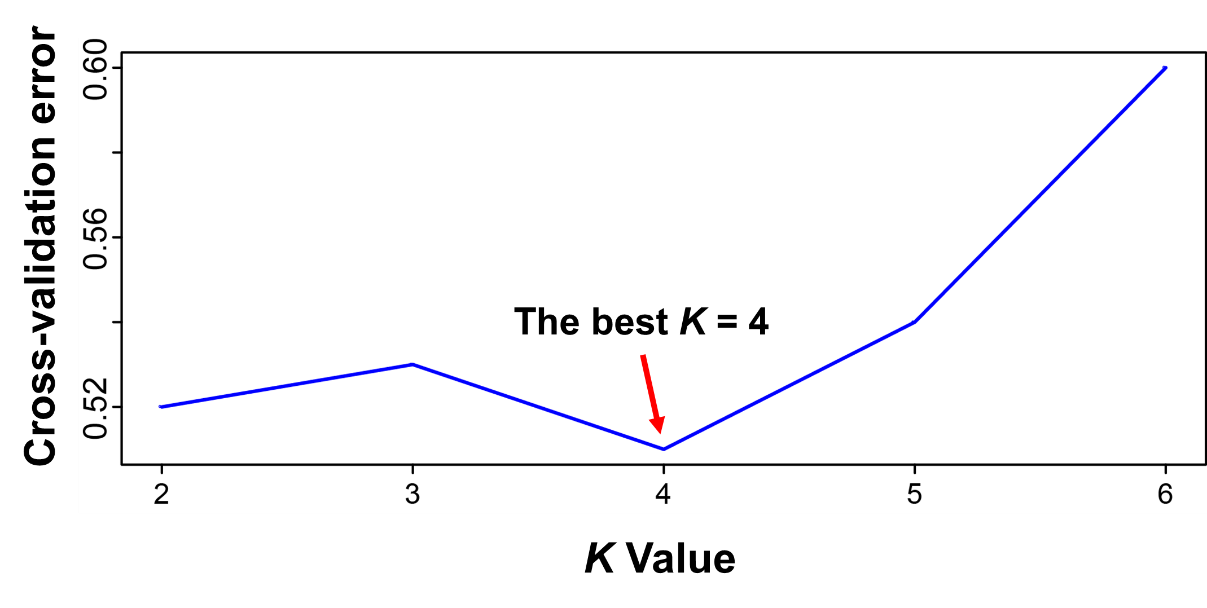


Fig. S4. Box plots of nucleotide diversity, calculated in 50 kb sliding window with 20 kb increments across the genome.


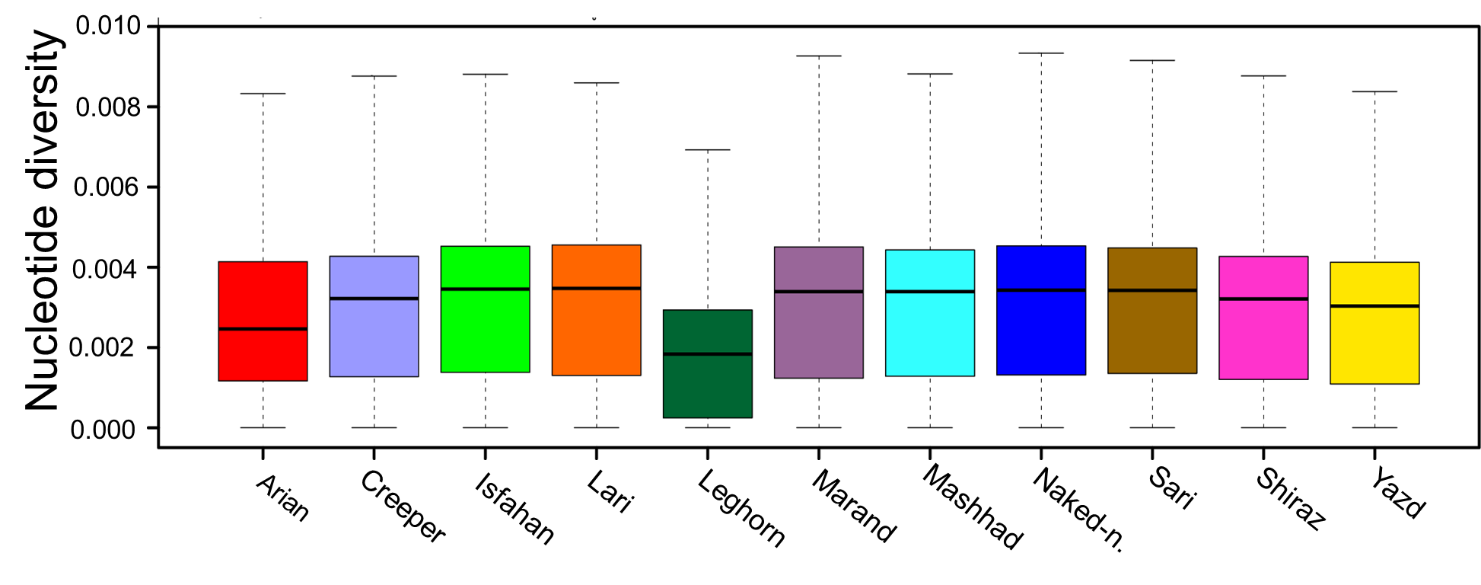


Fig. S5.Genomic regions with selection signals in native chickens by π method. (A) Indigenous group versus Arian chickens. (B) Indigenous group versus White Leghorn chickens.


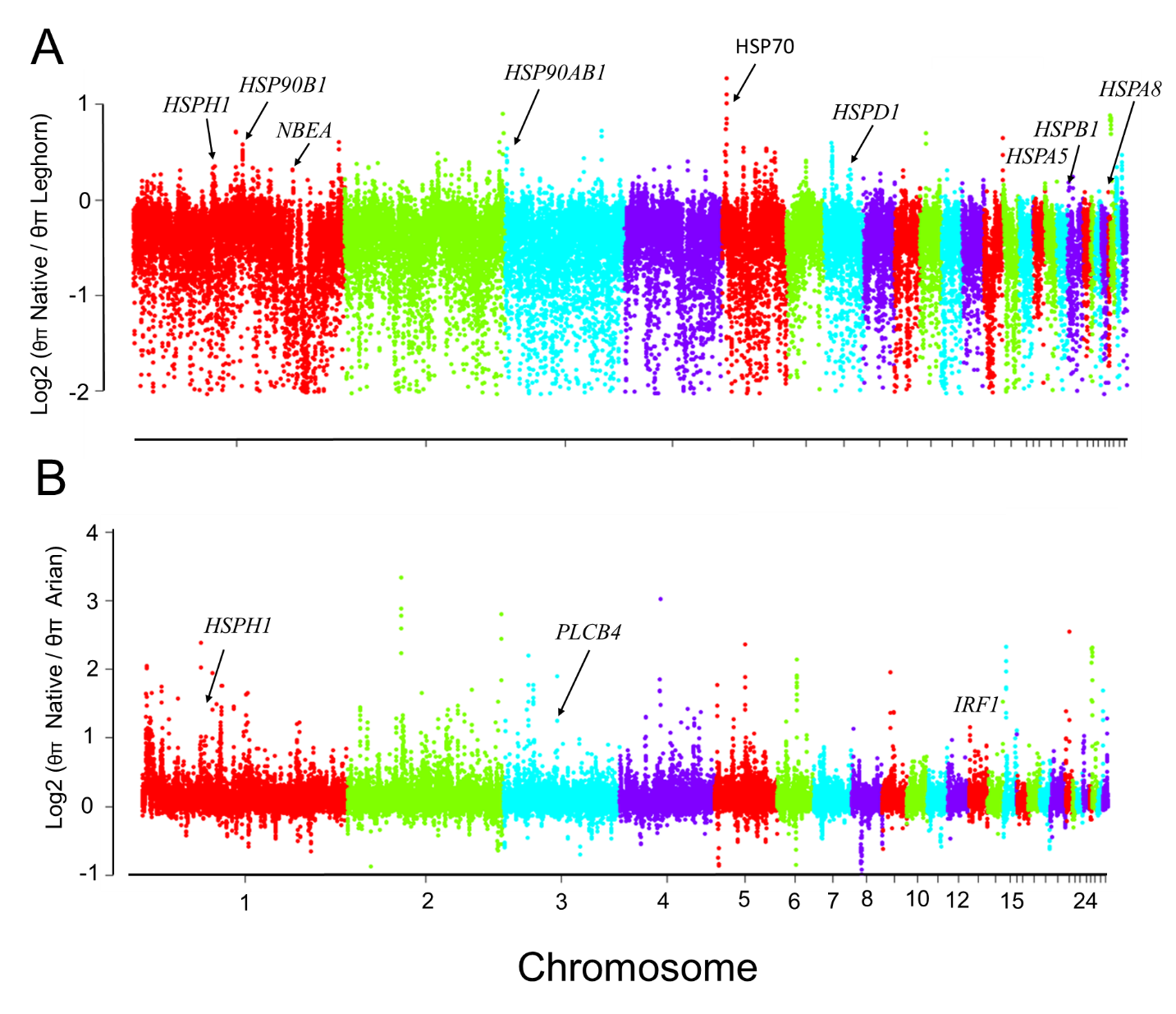


Fig. S6. Haplotype network based on pairwise differences within the selective sweep region (Chr1: 176.34-176.36) in *HSPH1*.


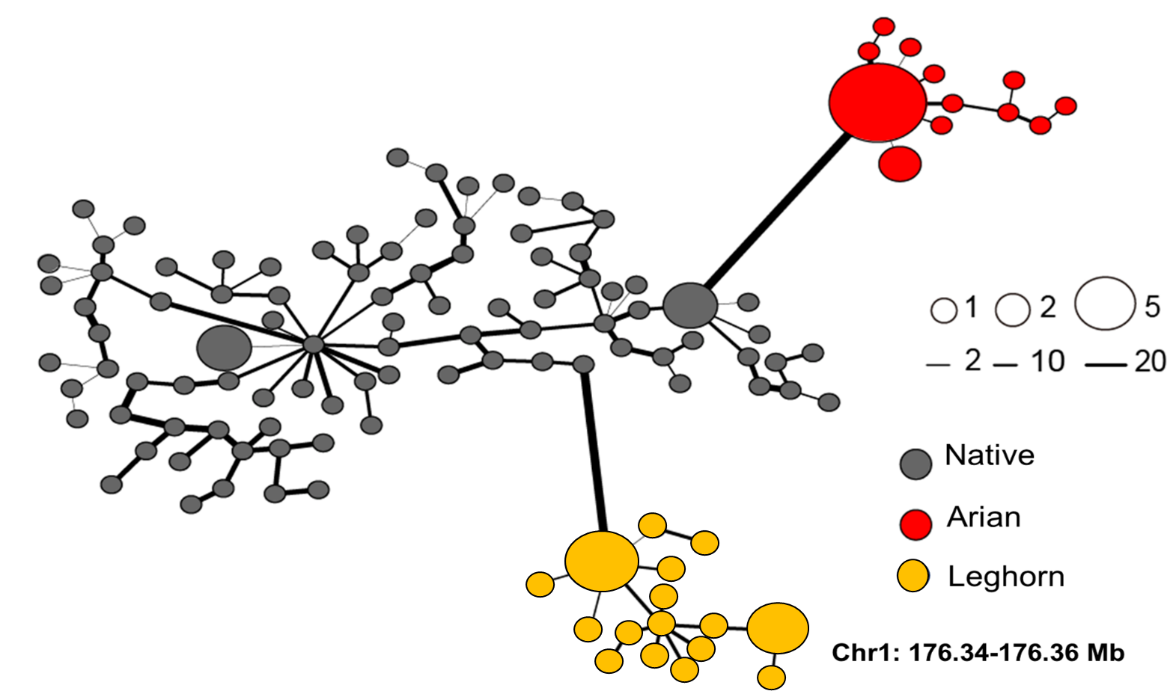

Supplement: Supplementary file 2 — Additional file 2: Figure S1.Venndiagrams summarize unique and common variants among groups. Fig. S2. Theheat map of ChromoPainter’s coancestry matrix. Each row corresponds to therecipient genomes and columns represent the donor individuals. Fig. S3. Cross validationerror (CV) plot from ADMIXTURE. Fig. S4. Box plots of nucleotide diversity, calculated in 50kb sliding window with 20 kb increments across the genome. Fig. S5. Genomic regions with selection signals in nativechickens by π method. (A) Indigenousgroup versus Arian chickens. (B) Indigenous group versus White Leghornchickens. Fig. S6. Haplotype network based on pairwise differenceswithin the selective sweep region (Chr1: 176.34-176.36) inHSPH1. [file 12864_2022_8434_MOESM2_ESM.docx]
